# Josephin Domain Structural Conformations Explored by Metadynamics in Essential Coordinates

**DOI:** 10.1371/journal.pcbi.1004699

**Published:** 2016-01-08

**Authors:** Marco A. Deriu, Gianvito Grasso, Jack A. Tuszynski, Diego Gallo, Umberto Morbiducci, Andrea Danani

**Affiliations:** 1 Istituto Dalle Molle di studi sull'Intelligenza Artificiale (IDSIA), Scuola universitaria professionale della Svizzera italiana (SUPSI), Università della Svizzera italiana (USI), Manno, Switzerland; 2 Department of Physics, University of Alberta, Edmonton, Alberta, Canada; 3 Department of Mechanical and Aerospace Engineering, Politecnico di Torino, Torino, Italy; University of Houston, UNITED STATES

## Abstract

The Josephin Domain (JD), i.e. the N-terminal domain of Ataxin 3 (At3) protein, is an interesting example of competition between physiological function and aggregation risk. In fact, the fibrillogenesis of Ataxin 3, responsible for the spinocerebbellar ataxia 3, is strictly related to the JD thermodynamic stability. Whereas recent NMR studies have demonstrated that different JD conformations exist, the likelihood of JD achievable conformational states in solution is still an open issue. Marked differences in the available NMR models are located in the hairpin region, supporting the idea that JD has a flexible hairpin in dynamic equilibrium between open and closed states. In this work we have carried out an investigation on the JD conformational arrangement by means of both classical molecular dynamics (MD) and Metadynamics employing essential coordinates as collective variables. We provide a representation of the free energy landscape characterizing the transition pathway from a JD open-like structure to a closed-like conformation. Findings of our *in silico* study strongly point to the closed-like conformation as the most likely for a Josephin Domain in water.

## Author Summary

Proteins are fascinating molecular machines capable of organizing themselves into well-defined hierarchical structures through a huge number of conformational changes to accomplish a wide range of cellular functions. Protein conformational changes may be characterized by transitions from a low-energy conformation to another. Computer simulations and in particular molecular modelling may be able to predict protein transition dynamics and kinetics, thus playing a key role in describing protein tendencies towards specific conformational rearrangements. Approaching this problem from an energetic point of view is of great importance especially in case of amyloidogenic proteins, given the intimate interconnection between the functional energy landscape and aggregation risk. In this work we have employed molecular modelling techniques to shed light into conformational dynamics and kinetics of the Josephin Domain, part of the protein Ataxin 3, which is responsible for the spinocerebbellar ataxia 3, also called Machado Joseph Disease. In greater detail, we have employed enhanced sampling approaches to provide an estimation of the free energy landscape characterizing the transition pathway among several known molecular arrangements of the Josephin Domain.

## Introduction

Proteins are fascinating molecular machines capable of organizing themselves into well-defined hierarchical structures through a huge number of conformational changes, in order to accomplish a wide range of cellular physiological functions. From an energy landscape point of view, protein conformational changes may be characterized by transitions from a low-energy conformation to another. In this connection, computational approaches have widely demonstrated their utility by providing important insights into the protein conformational features [1–5]. Molecular Dynamics simulations, and in particular enhanced sampling techniques, are able not only to predict protein transition pathways, but also to quantify the free-energy landscape along selected reaction coordinates, thus playing a key role in describing protein tendencies towards specific conformational rearrangements. Approaching this problem from an energetic point of view is of great importance especially in case of amyloidogenic proteins, given the intimate interconnection between the functional energy landscape and aggregation risk [6].

The Josephin Domain (JD), i.e. the N-terminal domain of Ataxin 3 (At3) protein, is an interesting example of competition between physiological function and aggregation risk [6,7]. In fact, the fibrillogenesis of Ataxin 3 is responsible for the spinocerebbellar ataxia 3, also called Machado Joseph Disease (MJD). Structurally, At3 is composed of a structured globular N-terminal region (i.e. the JD, residues Met1-Arg182 in the human protein), combined with a more flexible C-terminal tail that contains the polyQ tract and the Ubiquitin Interacting Motifs (UIM) [8,9].

The expansion of polyglutamine (polyQ) tract in Ataxin 3 (so-called expanded At3) is considered a cause for protein misfolding and aggregation, but the underlying mechanism remains to be elucidated.

Although it is known that the polyQ tract is necessary for kinetic instigation of an aggregation mechanism [10–14], several experimental studies support the hypothesis that JD structural stability could play a major role in determining the aggregation features and toxicity of polyQ proteins [7,15–21].

In this regard, experimental evidences have suggested a two-stage pathway for At3 fibrillogenesis: the first, JD-mediated and the second, polyQ-dependent [19,22,23]. Fibrillar aggregates of both not-expanded At3 and isolated JD have shown markedly similar morphological and mechanical properties, suggesting a leading role for the JD in the mechanism of fiber formation [17]. Moreover, inhibition of JD self-association by a small heat-shock protein significantly slows down expanded At3 aggregation [24]. For these reasons, the role of JD has been the subject of a robust debate in the past [6,7,18–21,25,26].

To date, several JD models solved by NMR are available in the literature (PDB entry 2JRI [27], 1YZB [28], 2AGA [29] and 2DOS [30]—UNIPROTID: P54252). Differences in the available models are located in the hairpin region (region α2-α3, residues Val31-Leu62). In particular, the 1YZB and 2JRI models are characterized by a “half-open” and “open” L-shape hairpin conformation, respectively. On the other hand, the “closed” 2AGA and “half-closed” 2DOS models exhibit the hairpin region packed against the rest of the globular structure [30]. Whereas all the above-mentioned NMR data have demonstrated the existence of several different available conformations for the JD, issues concerning *i*) the likelihood of JD achievable conformational states in solution, and *ii*) the role played by environmental conditions (such as the solution’s pH) and interacting physiological partners (such as ubiquitin) in JD conformational arrangement are still unresolved. Specifically, results from a recent characterization of the JD free energy landscape using MD simulations suggested the open-like model as the most representative of the JD structure in solution [31]. Nevertheless, other previous experimental and computational studies strongly support the idea that JD has a highly flexible extended hairpin in dynamic equilibrium between open and closed states [1,30]. In a very recent *in silico* study, the early stage of the JD-JD dimerization mechanism [1] has been investigated by MD and indicates that the JD-JD binding might play a role in determining the kinetics of hairpin opening/closure. However, the previous computational investigation is limited to a classical MD approach with a relatively short simulation timescale [1].

In principle, to prove the JD open-like or the JD closed-like configuration as favored, it would be necessary to show not only that *i*) there is more sampling in one state during a classical MD, but also that *ii*) several transitions between states are sampled during the simulation. Hence, an accurate evaluation of the JD conformational changes requires a longer simulation time-scale and robust sampling methods. In this regard, enhanced sampling methods represent a powerful tool to improve the sampling efficiency of classical MD [32–40], including those that artificially add an external driving force to guide the protein from one structure to another [38,41]. Moreover, reducing the dimensionality of the trajectory obtained from MD simulations can help identify the dominant modes in the motion of the molecule [38,41].

Motivated by the still open debate regarding the most representative JD structural arrangement [1,30,31,42,43], we have carried out an investigation on JD conformational changes using both unbiased MD and Metadynamics guided by essential coordinates. In this work, we provide an estimation of the free energy landscape characterizing the transition pathway from a JD open-like to a closed-like structure (which is henceforth called the folding pathway). The findings of our *in silico* study strongly suggest the closed conformation as the most likely for a Josephin Domain in water.

## Materials and Methods

### Classical Molecular Dynamics and Principal Component Analysis

The 1YZB model [28,42] of JD was selected as the starting point for the present work. The rationale for this choice is in the experimental work of Nicastro et al. [42] indicating a satisfactory validation of the 1YZB model through the application of an arsenal of tools for checking the quality, accuracy and mutual consistency of the structures available.

The 1YZB model was solvated in a dodecahedron box where the minimum distance between the protein and the edge of the box was 1 nm, resulting in a molecular system of about 50,000 interacting particles. The net charge of the system was neutralized by the addition of Cl^−^ and Na^+^ ions. AMBER99-ILDN force-field [44–46] and water TIP3P model [47] were chosen to describe the system’s topology. Particle-Mesh Ewald method with a short-range cut off of 1.2 nm was applied to treat electrostatics. A cut-off of 1.2 nm was also applied to Lennard-Jones interactions.

The system was minimized by the steepest descent energy minimization algorithm (1000 steps). Then, in order to increase the statistics of MD data, five replicas, differing in initial atom velocities, were created from the minimized system. In particular, for each replica, a random velocity taken from a Maxwell-Boltzmann distribution at 310 K was assigned to every atom of the system (i.e. JD, water and ions). A position-restrained and production MD simulations were carried out as described in the following. Two subsequent MD simulations (500 ps, and 100 ps, respectively) were run in the NPT ensemble, applying position restraints of 1000, and 100 kJ/mol/nm^2^, respectively, to the JD *Cα* atoms. System temperature was set at 310 K by using the *v-rescale* [48] thermostat with a coupling time step of 0.1 ps. Moreover, in NPT simulations a *Berendsen* barostat [49] was also employed with a reference pressure of 1 atm and a coupling time step of 1.0 ps. A third position restrained dynamics simulation (100 ps) was carried out by applying a force constant of 10 kJ/mol/nm^2^in the NVT ensemble at 310 K. Finally, an unrestrained production MD of 500 ns was run in the NVT ensemble at 310 K, as done in several previous Molecular Dynamics studies [50–52]. GROMACS 4.6 package was employed for all MD simulations and data analysis [53]. Ensemble data taken from all production MD trajectories of the above-mentioned five replicas (each simulated for 500 ns) were used for JD conformational analysis. The Visual Molecular Dynamics (VMD) package [54] provided the visual inspection of the simulated systems. Dedicated GROMACS tools were used for quantitative analyses in terms of Root-Mean-Square Deviation (RMSD) and Root-Mean-Square Fluctuation (RMSF). The secondary structure of the protein has been calculated by the STRIDE software [55] on several snapshots along the simulation time.

The identification of JD conformational transitions from open to closed JD conformations has been carried out by employing quantities which have already been demonstrated to be meaningful in describing the JD transition pathway: the radius of gyration (RG) and the hairpin angle [1,31].

Given that the NMR models (1YZB, 2JRI, 2DOS, and 2AGA) considered in this work present a different number of residues, the RG has been calculated by considering all the residues in common among the above mentioned PDB models. In detail, the residue range 1Met-171Asp (according to 1YZB numbering) has been chosen.

The hairpin angle was calculated from the centers of mass of the *Cα* atoms from three distinct JD regions: globular subdomain (residues 111–113, 122–125 and 162–165), hinge (residues 32–35) and loop (residues 45–48 and 58–61) [31] (Section 3 in S1 Text).

Principal Component Analysis (PCA) was applied to classical MD trajectories. PCA is an established method which allows to elucidate large-scale and low-frequency modes, respectively, yielding collective motions directly related to a specific molecular event [56]. In detail, after the alignment of the JD *Cα* Cartesian coordinates, the covariance matrix was calculated and diagonalized (Section 2 in S1 Text).

### Metadynamics

The free energy landscape representing the JD folding pathway was investigated by means of Metadynamics [58,59], a powerful technique for enhancing the sampling in MD simulations and reconstructing the free-energy surface as a function of few selected collective variables (CVs). The first eigenvector derived from the PCA was used as CV for a well-tempered Metadynamics simulation of 500 ns starting from the open-like 1YZB model [16]. The JD model was prepared for Metadynamics by applying system minimization and position restraint dynamics, as described above for the classical MD.

To perform Metadynamics simulations, a Gaussian width of 0.1 was used. Along the simulation, the initially prescribed Gaussian deposition rate value of 0.2 kJ/mol·ps was used and it gradually decreased on the basis of an adaptive scheme, with a bias factor of 20. The setting of Gaussian width and deposition rate was done on the basis of a well-established scheme [37,40]. In particular, the Gaussian width value was of the same order of magnitude as the standard deviation of the collective variable, calculated during unbiased simulations (production MD). The authors have also verified that the maximum force introduced by a single Gaussian distribution is smaller than the typical derivative of the free energy.

The estimation of the free energy profile was performed by employing the reweighted-histogram procedure [60,61], taking into account for the following collective variables: the projection along the first PCA eigenvector, the JD’s RG, the hairpin angle and the alphaRMSD variable. More specific information about the definition of the CVs, the convergence of the Metadynamics simulations and the free energy reconstruction is reported in Section 3 in S1 Text. GROMACS 4.6 package patched with PLUMED was employed for metadynamics simulations and data analysis [57].

## Results

### Classical Molecular Dynamics and Mode Analysis

As stated above, five independent replicas of a single JD in explicitly modeled water and ions were simulated for 500 ns. Structural conformational properties and stability were initially checked by monitoring the time evolution of the RMSD and secondary structure (Section 1 in S1 Text). The data generated indicated that a reasonable stability of the above-mentioned quantities has been reached in all cases in the last 100 ns of the production run of the MD simulations. Moreover, the JD secondary structure showed to be highly conserved throughout the whole simulation time (Section 1 in S1 Text).

**Fig 1:**
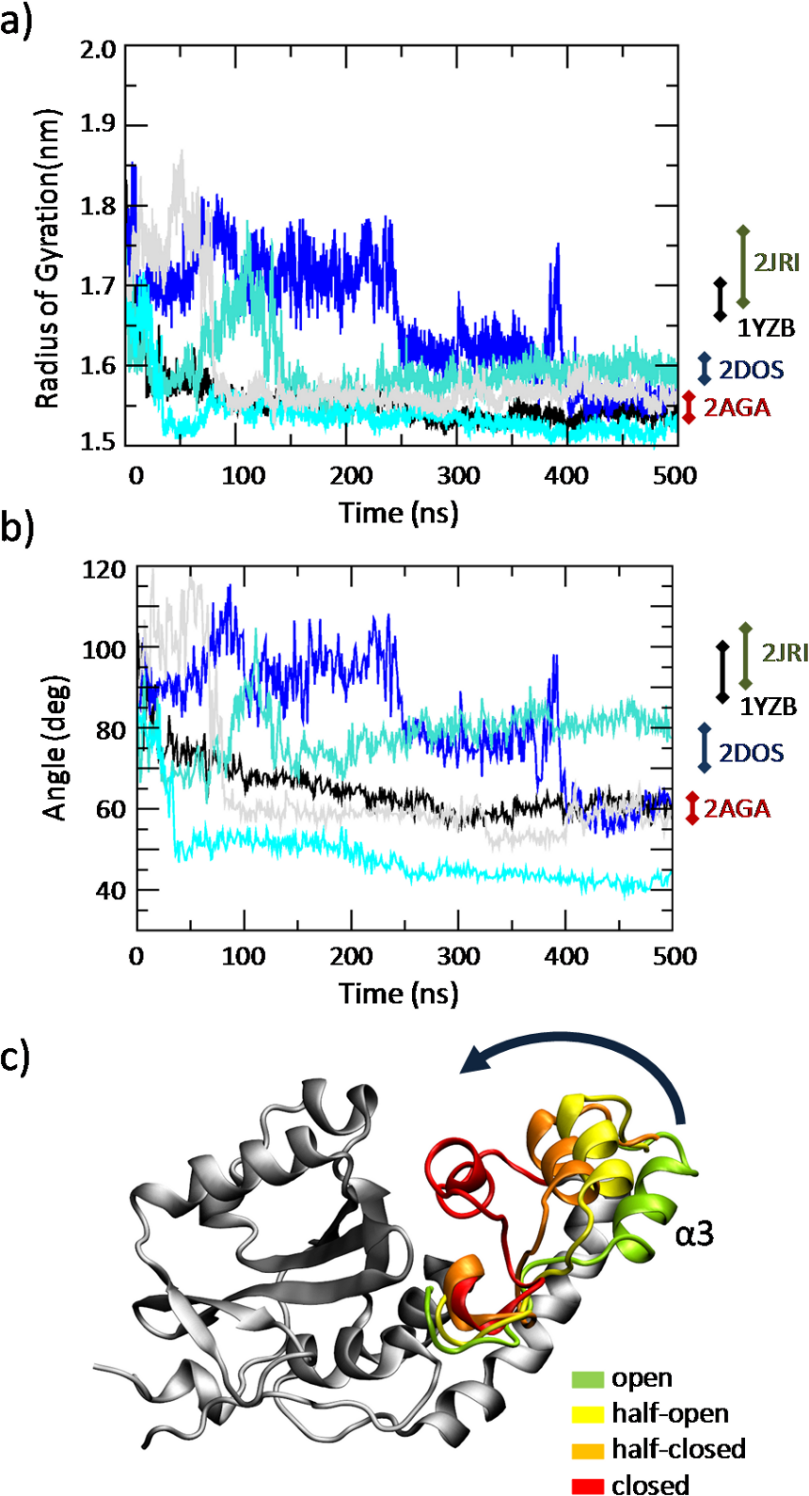
Time evolution of the JD radius of gyration (a) and hairpin angle (b) throughout the MD trajectory of each replica. (c) Visual inspection of the JD conformations taken from the classical MD simulations. The MD trajectories reveal a JD transition from an open state to a closed state, through an half-open and half-closed state. For each snapshot the α3 region (Asp57-Leu62) is highlighted with a different color: green (open), yellow (half-open), orange (half-closed) and red (closed).

The time evolution of the RG calculated over the classical MD trajectories (Fig 1A) reveals the JD transition for all replicas, from a half-open (starting configuration 1YZB) to a closed or half-closed conformation, characterized by RG lower than 1.6 nm (Fig 1A) and a hairpin angle lower than 80° (Fig 1B). Several intermediate half-open and half-closed conformations are explored during the MD simulation (Fig 1C). Moreover, no transition from the reached JD closed-like to the open-like structure has been detected during the simulated time.

**Fig 2:**
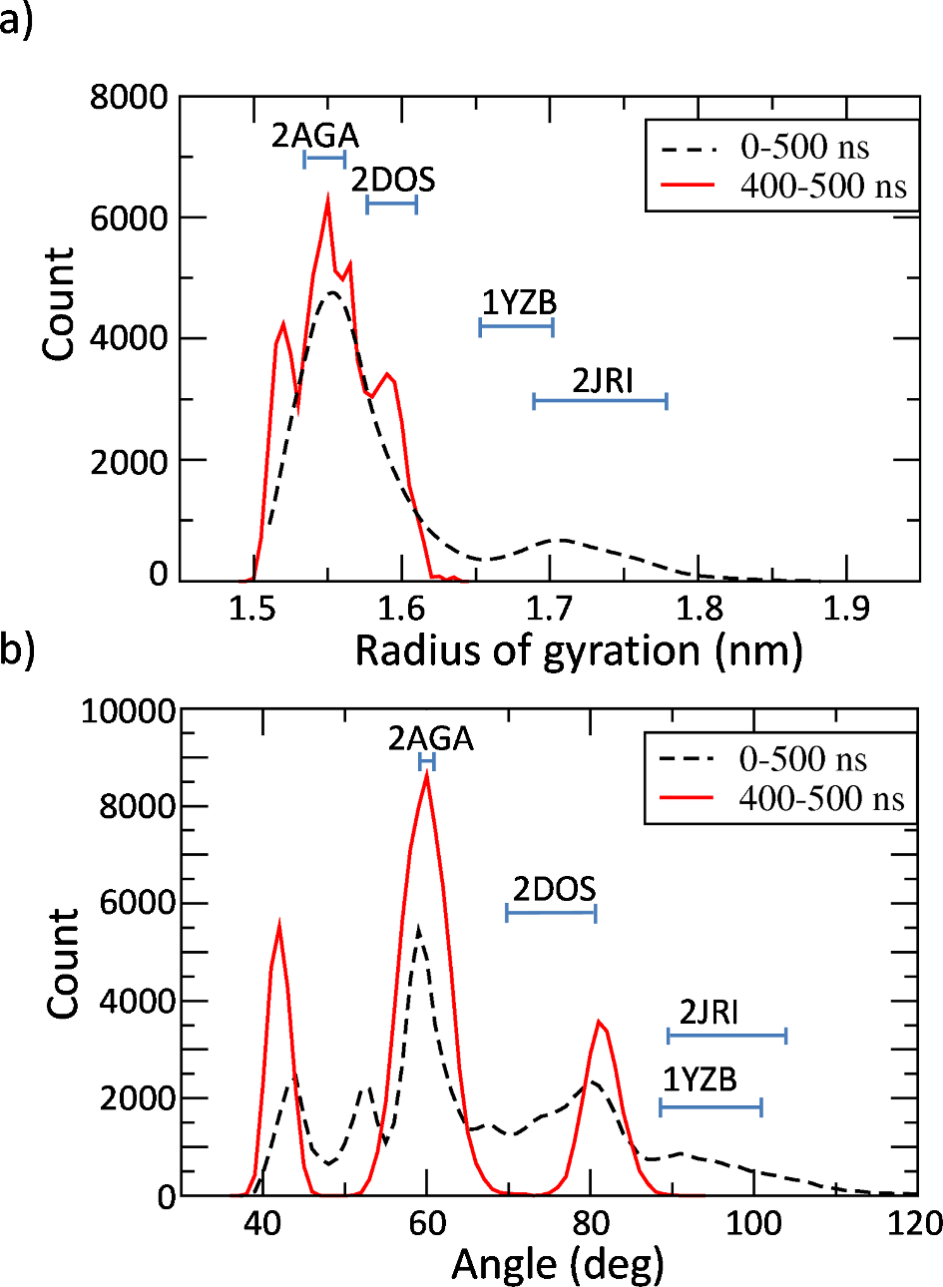
Distribution of JD radius of gyration (a) and hairpin angle (b) calculated for the entire MD trajectories (black dashed curve) and at the stability (from 400 to 500 ns, red line). MD trajectories of the five replicas are used as ensemble data. Cyan bars indicate the value range of RG (a) and hairpin angle (b) calculated by NMR available data of 2JRI, 1YZB, 2DOS, and 2AGA.

By analyzing the same data in the form of a distribution plot (Fig 2), it is possible to observe that sampled structures in the stability region of the simulation (400–500 ns) are far from open like JD arrangement.

Secondly, it is worth mentioning the different distribution shape when considering the whole trajectory (0–500 ns, black dashed line in Fig 2) and the trajectory in the stability region (400–500 ns, red line in Fig 2). Specifically, a curve comparison of both the RG and hairpin angle distribution indicates how intermediate states have a tendency to converge toward closed like arrangements. The importance of using both the RG and hairpin angle to analyze the JD arrangement is demonstrated by looking at the NMR range values labeled in Fig 2. Namely, whereas the RG helps in discerning between half-open (1YZB) and open (2JRI) JD arrangement (Fig 2A) the hairpin angle perfectly distinguish between closed (2AGA) and half-closed (2DOS) JD (Fig 2B).

**Fig 3:**
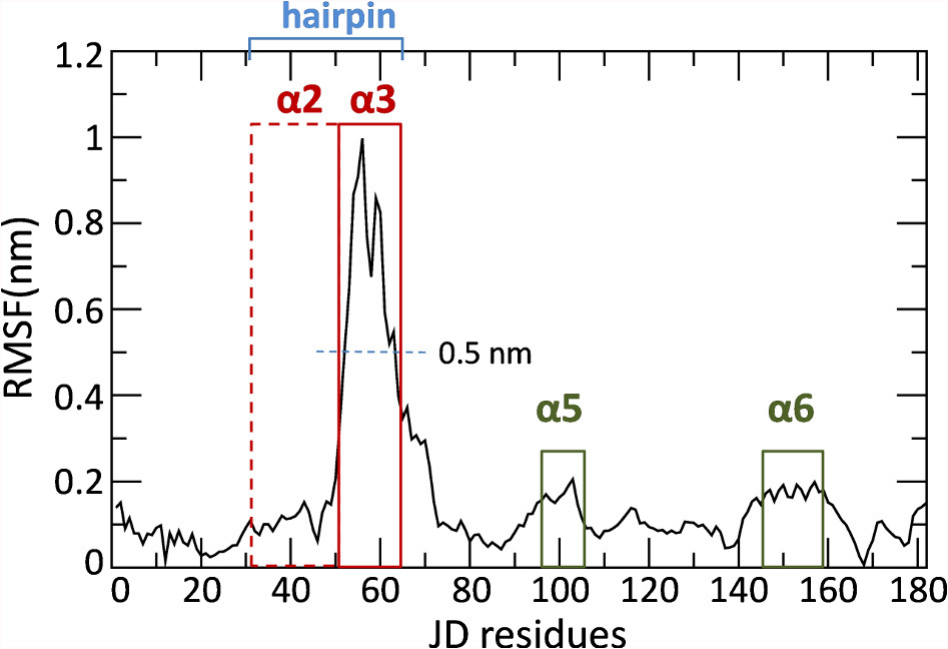
Root Mean Square Fluctuation plot. **Each point represents the mean fluctuation for each JD residue calculated over the whole MD trajectory (500 ns and five replicas taken together) filtered on the first PCA eigenvector.** The hairpin region (Val31-Leu62), composed by helix α2 (dashed red line) and α3 (continuous red line) is highlighted in blue. Secondary structure α6 (Asp145-Glu158) is highlighted in dark green.

To reduce the high-dimensionality of the MD trajectory and to identify the dominant molecular phenomena related to the hairpin closure, PCA was applied. After the alignment of the JD *Cα* atoms, the MD trajectory was filtered to show only the motion along the first eigenvector, calculated by covariance matrix diagonalization. More information on PCA and the eigenvector values is reported in Section 2 in S1 Text. The JD Root Mean Square Fluctuation (RMSF) calculated over the filtered trajectory (Fig 3) shows that, as expected, the first PCA eigenvector effectively captures the hairpin motion (RMSF_hairpin_>0.5 nm).

### Metadynamics

**Fig 4:**
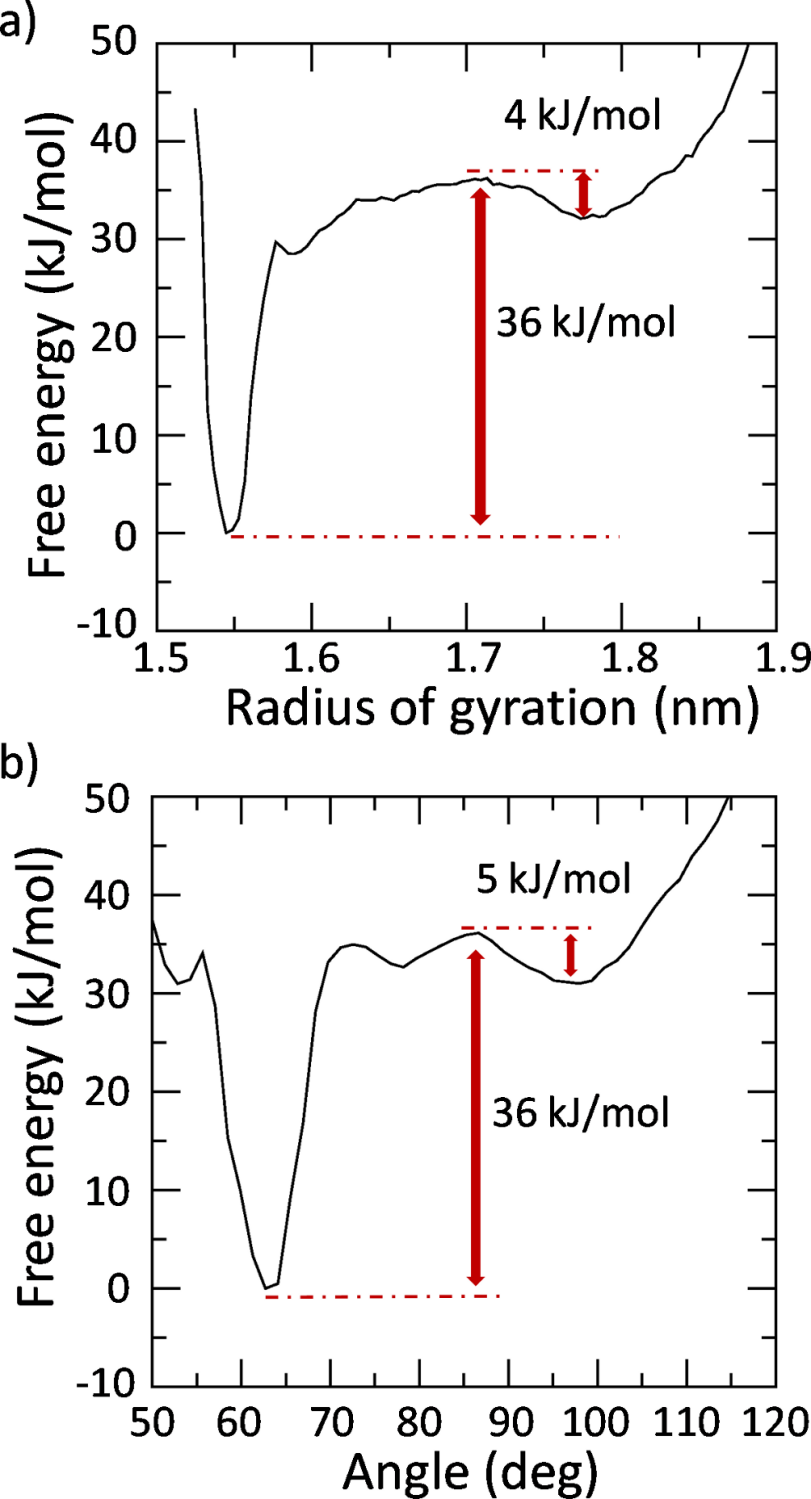
Free energy profiles of the JD transition pathway as function of the (a) radius of gyration (RG) and (b) hairpin angle. The depth of the energy well corresponding to the absolute free energy minimum is highlighted in red. The absolute free energy well is set as zero.

The first eigenvector derived from the PCA was used as collective variable (CV) for a well-tempered Metadynamics simulation. Analyzing the free energy profiles reported in Fig 4A as a function of the RG, two energy wells of 36 kJ/mol and 4 kJ/mol, located at RG values of 1.55 nm and 1.78 nm, respectively, can be identified. This result is also confirmed by reweighting the free energy profile as function of the hairpin angle (Fig 4B). In this case, the deepest free energy minimum (36 kJ/mol) is found to be located at a value of the hairpin angle equal to 63°. A second minimum (5 kJ/mol) is found to be located at a value of the hairpin angle equal to 100°.

As expected, the RG and hairpin angle values corresponding to the free energy wells (Fig 4A) are in agreement with the distribution peaks obtained from the unbiased MD simulations in the stability region (400–500 ns) shown in Fig 2. This finding confirms the reliability of our Metadynamics results given that the free energy minima are expected to identify the most energetically favorable configuration.

**Fig 5:**
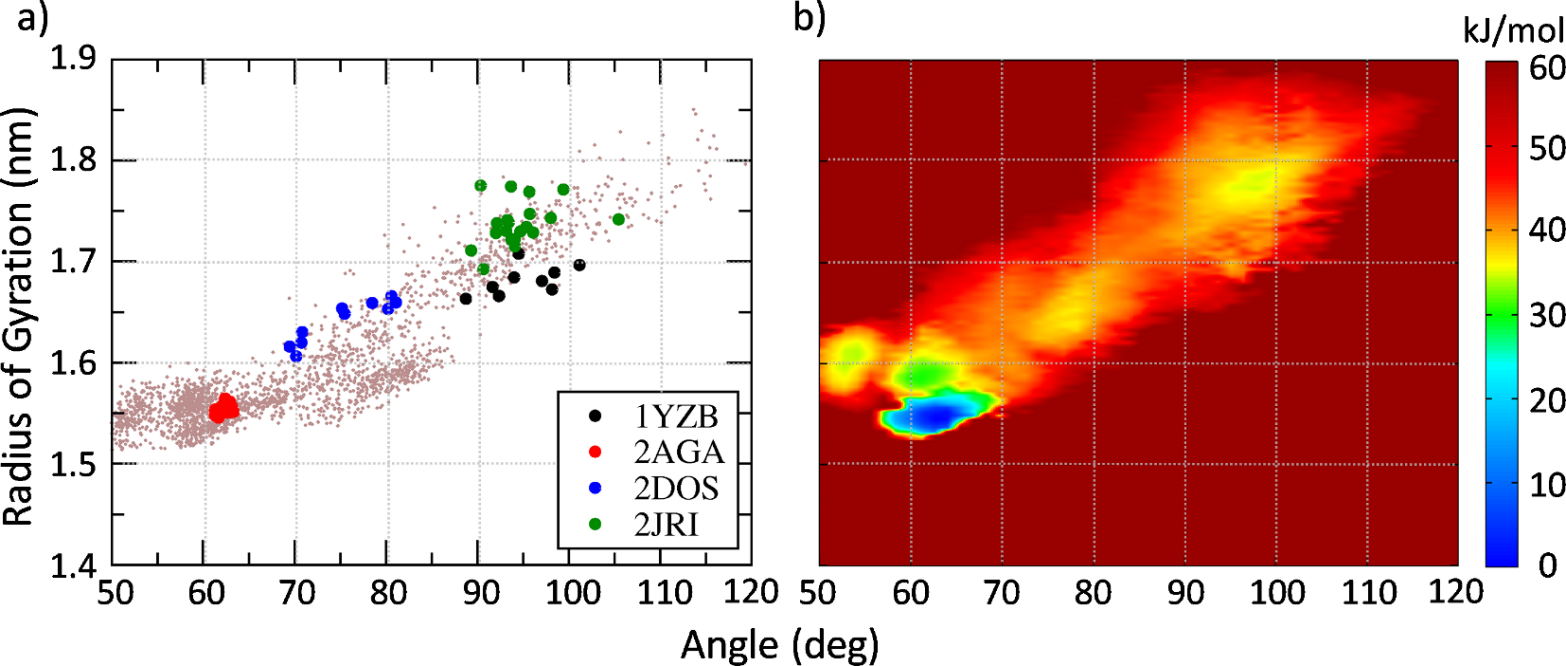
(a) Each point (colored in pink) represents a snapshot identified by the radius of gyration (y axis) and the hairpin angle (x axis) for the entire MD conformational ensemble sampled by classical MD (5 replicas of 500 ns). The hairpin angle and radius of gyration of all NMR snapshots derived from the JD models available in literature are colored in red (2AGA), blue (2DOS), black (1YZB) and green (2JRI). (b) 2D color map of the JD free energy landscape, calculated by the reweighting histogram approach on the Metadynamics outcome. The free energy values are represented as function hairpin angle and JD radius of gyration. The absolute free energy well is set as zero.

An overall picture of the JD free energy landscape is provided in Fig 5B, showing the 2D color map of the free energy profile as a function of the RG and hairpin angle. Again, the free energy minima are expected to match the most energetically favorable JD configurations. Hence, it is interesting to compare the free energy map with the JD configurations sampled by classical MD (Fig 5A). Fig 5A also provides the snapshots derived from the JD models available in the literature. Interestingly, 2AGA, 2DOS and 2JRI models lie in regions regularly sampled by classical MD, and characterized by absolute or relative free energy minima. The most sampled configurations by classical MD, corresponding in term of RG and hairpin angle to the 2AGA model, is also the deepest energy minimum in the free energy landscape. Similar characteristics between metadynamics lowest energy state and the 2AGA model are also highlighted by contact maps reported in Section 2 in S1 Text. On the contrary, values of the RG and hairpin angle corresponding to the 1YZB, i.e., starting structure of our simulations, are merely sampled by classical MD and far from the absolute energy minimum in Metadynamics.

## Discussion

The characterization of the free energy landscape in a protein folding pathway represents a significant contribution to both experimental and theoretical approaches, given the intimate interconnection between the functional energy landscape and aggregation risk [6].

The JD folding pathway is an issue still debated in the literature [30,31,42] since a decisive proof of the most likely JD conformation has not been provided yet. Several JD models, solved by NMR, are available in the literature: open (2JRI) [27], half-open (1YZB [28], closed (2AGA) [29] and half-closed (2DOS) [30]. The above mentioned models have been questioned and debated in the recent literature on both computational and experimental studies. In agreement with previous research in this area [30] we have recently observed the metastable behavior of the JD, which is dynamically able to switch between an open-like and closed-like structure during the dimerization [1]. However, in order to predict the JD free energy landscape, the conformational ensemble provided by the classical MD simulation is not adequate. In particular, data from MD with a timescale of hundreds of ns show that the JD closed-like state is achievable starting from an open-like state whereas a transition from a completely closed-like to an open-like state has never be detected (Fig 1 and Section 1 in S1 Text). The classical MD simulation sampling is in this case insufficient, because when the system is trapped in the energy minimum characterized by the closed-like structure, thermal fluctuations are not enough to overcome the energy barrier (around 36 kJ/mol) needed to switch to an open-like configuration. A classical MD simulation might never be able to get out of such a deep energy minimum. To overcome this limitation, inherent to the classic MD, Metadynamics can be used to sample large-scale protein transitions as demonstrated by some relevant and pioneering papers in the field [62–64].

Our work brings together the efficient sampling of Metadynamics with a MD-PCA-based dimensionality reduction method. In particular, PCA was used to elucidate the transition pathway between the JD open-like and closed-like models, and Metadynamics was performed to estimate the corresponding free-energy landscape. Nevertheless, this computational approach was already successfully applied earlier [38,65–69], thus confirming its promise as a successful strategy for investigating conformational changes in complex biomacromolecules.

A limitation of the presented approach is, in fact, that preliminary information regarding the molecular transition is needed in order to calculate the essential coordinates: the transition from a JD open-like to a closed-like conformation allowed us to obtain the CV to guide the Metadynamics method.

Our findings confirm that the JD hairpin region, which protrudes out into the solvent, can be responsible for an extensive conformational change, switching between the open-like conformation and the closed-like one. As suggested in previous works [1,30], the hairpin mode of motion mainly consists of movement of region α3 (Asp57-Leu62) toward α6 (Asp145-Glu158) (Figs 1C and 3).

Interestingly, in our simulations, when the JD is alone in water environment the most stable configuration is characterized by the hairpin packed against the globular core, in agreement with models 2AGA [29] (Figs 4 and 5). However, an energy well of about -5 kJ/mol has been also detected corresponding to the open conformation, in agreement with the 2JRI [27] model. In general, such free energy minima should predict the most favorable conformational state. In fact, the RG values corresponding to the free energy minima are consistent with the peaks of the RG distribution of the unbiased simulation at equilibrium (Figs 1, 4 and 5), indicating the reliability of the presented approach in describing the JD free energy landscape. Surprisingly the JD half-open structure, with the RG and hairpin angle mainly corresponding to the 1YZB is the less sampled structure even during classical MD simulations.

In this connection, it is important to clarify that our work is not oriented to evaluate the “quality” of produced NMR which has already been checked with proper methodologies [42]. Instead, a first novel aspect of our work is that the employed approach has demonstrated to explore the state space by granting several transitions among closed and open JD conformations.

Data reported in recent literature [31] indicated the JD open structure (particularly referring to the 1YZB model) as the most likely JD conformation in water. Moreover, it has been emphasized how the 2AGA ensemble data were far away from the calculated free energy minima [31]. However, it is worth mentioning that, all the previous computational works [1,31,43] did not show transitions between open and closed JD and *viceversa*. In our opinion, such transitions are required to claim that a specific protein conformational state is characterized by lower free energy minima than another one. In fact, in the partial section of the free energy profile corresponding to the JD open-like structure, our results are in agreement with the above mentioned recent work [31]. However, the representation of the whole free energy profile describing both open and closed structures demonstrates the closed arrangement as the most likely for a Josephin Domain in water environment.

Nonetheless, there may be conditions under which the open-like state is stabilized (e.g. in the context of the full-length protein or in the presence of a physiological partner). For example, we have recently shown how the JD conformational state might be affected by the presence of another interacting JD [1] as well as by an inorganic surface [43]. In addition, as already suggested [30], the JD free energy landscape could be influenced by other environmental conditions, such as temperature and pH.

Further investigations are planned and will help in clarifying the influence of JD functional partners and environmental factors affecting the JD conformational arrangement. This information may be relevant not only to better understand the physiological function of the Josephin Domain, but also to provide insight into molecular phenomena characterizing the pathological nature of spinocerebellar ataxia 3.

## Supporting Information

S1 Text The file contains: data analysis on Classical Molecular Dynamics trajectories and Metadynamics Lowest Energy States (Section 1); details on Principal Component Analysis (Section 2); procedures and validation checks on Metadynamics simulations (Section 3). (PDF)

## References

[1] Deriu MA, Grasso G, Licandro G, Danani A, Gallo D, Tuszynski JA, et al Investigation of the josephin domain protein-protein interaction by molecular dynamics. Salahub D, editor. PLoS One. Public Library of Science; 2014;9: e108677 10.1371/journal.pone.0108677 25268243 PMC4182536

[2] Deriu MA, Shkurti A, Paciello G, Bidone TC, Morbiducci U, Ficarra E, et al Multiscale modeling of cellular actin filaments: from atomistic molecular to coarse-grained dynamics. Proteins. 2012;80: 1598–609. 10.1002/prot.24053 22411308

[3] Deriu MA, Bidone TC, Mastrangelo F, Di Benedetto G, Soncini M, Montevecchi FM, et al Biomechanics of actin filaments: a computational multi-level study. J Biomech. Elsevier; 2011;44: 630–6. 10.1016/j.jbiomech.2010.11.014 21130998

[4] Pavan GM, Danani A. Dendrimers and dendrons for siRNA binding: computational insights. J Drug Deliv Sci Technol. 2012;22: 83–89. 10.1016/S1773-2247(12)50008-0

[5] Pavan GM, Mintzer MA, Simanek EE, Merkel OM, Kissel T, Danani A. Computational insights into the interactions between DNA and siRNA with “rigid” and “flexible” triazine dendrimers. Biomacromolecules. 2010;11: 721–30. 10.1021/bm901298t 20131771 PMC3841066

[6] Gershenson A, Gierasch LM, Pastore A, Radford SE. Energy landscapes of functional proteins are inherently risky. Nat Publ Gr. Nature Publishing Group; 2014;10: 884–891. 10.1038/nchembio.1670 PMC4416114 25325699

[7] Laura M, Giuseppe N, Lesley C, Michele V, Annalisa P, Masino L, et al Functional interactions as a survival strategy against abnormal aggregation. FASEB J. 2011;25: 45–54. 10.1096/fj.10-161208 20810784 PMC3005437

[8] Takiyama Y, Nishizawa M, Tanaka H, Kawashima S, Sakamoto H, Karube Y, et al The gene for Machado–Joseph disease maps to human chromosome 14q. Nat Genet. 1993;4: 300–304. 10.1038/ng0793-300 8358439

[9] Kawaguchi Y, Okamoto T, Taniwaki M, Aizawa M, Inoue M, Katayama S, et al CAG expansions in a novel gene for Machado-Joseph disease at chromosome 14q32. 1. Schulenberg T, Ozawa M, Grotzbach G, editors. Nat Genet. Forschungszentrum Karlsruhe; 1994;8: 221–228. 10.1038/ng1194-221 7874163

[10] Dürr A, Stevanin G, Cancel G, Duyckaerts C, Abbas N, Didierjean O, et al Spinocerebellar ataxia 3 and Machado-Joseph disease: clinical, molecular, and neuropathological features. Ann Neurol. 1996;39: 490–9. 10.1002/ana.410390411 8619527

[11] Ranum LP, Lundgren JK, Schut LJ, Ahrens MJ, Perlman S, Aita J, et al Spinocerebellar ataxia type 1 and Machado-Joseph disease: incidence of CAG expansions among adult-onset ataxia patients from 311 families with dominant, recessive, or sporadic ataxia. Am J Hum Genet. 1995;57: 603–608. Available: http://www.pubmedcentral.nih.gov/articlerender.fcgi?artid=1801263&tool=pmcentrez&rendertype=abstract 7668288 PMC1801263

[12] Zoghbi HY, Orr HT. Glutamine repeats and neurodegeneration. Annu Rev Neurosci. 2000;23: 217–47. 10.1146/annurev.neuro.23.1.217 10845064

[13] Maciel P, Gaspar C, DeStefano AL, Silveira I, Coutinho P, Radvany J, et al Correlation between CAG repeat length and clinical features in Machado-Joseph disease. Am J Hum Genet. 1995;57: 54–61. Available: http://www.pubmedcentral.nih.gov/articlerender.fcgi?artid=1801255&tool=pmcentrez&rendertype=abstract 7611296 PMC1801255

[14] Riess O, Rüb U, Pastore A, Bauer P, Schöls L. SCA3: neurological features, pathogenesis and animal models. Cerebellum. 2008;7: 125–37. 10.1007/s12311-008-0013-4 18418689

[15] L. Robertson A, P. Bottomley S. Towards the Treatment of Polyglutamine Diseases: The Modulatory Role of Protein Context. Curr Med Chem. 2010;17: 3058–3068. 10.2174/092986710791959800 20629626

[16] Masino L, Nicastro G, Menon RP, Dal Piaz F, Calder L, Pastore A. Characterization of the structure and the amyloidogenic properties of the Josephin domain of the polyglutamine-containing protein ataxin-3. J Mol Biol. 2004;344: 1021–35. 10.1016/j.jmb.2004.09.065 15544810

[17] Masino L, Nicastro G, De Simone A, Calder L, Molloy J, Pastore A. The Josephin domain determines the morphological and mechanical properties of ataxin-3 fibrils. Biophys J. Biophysical Society; 2011;100: 2033–2042. 10.1016/j.bpj.2011.02.056 PMC3077691 21504740

[18] Nicastro G, Masino L, Esposito V, Menon RP, De Simone A, Fraternali F, et al Josephin domain of ataxin-3 contains two distinct ubiquitin-binding sites. Biopolymers. 2009;91: 1203–14. 10.1002/bip.21210 19382171

[19] Ellisdon AM, Thomas B, Bottomley SP. The two-stage pathway of ataxin-3 fibrillogenesis involves a polyglutamine-independent step. J Biol Chem. 2006;281: 16888–96. 10.1074/jbc.M601470200 16624810

[20] Chow MKM, Paulson HL, Bottomley SP. Destabilization of a Non-pathological Variant of Ataxin-3 Results in Fibrillogenesis via a Partially Folded Intermediate: A Model for Misfolding in Polyglutamine Disease. J Mol Biol. 2004;335: 333–341. 10.1016/j.jmb.2003.08.064 14659761

[21] Natalello A, Frana AMA, Relini A, Apicella A, Invernizzi G, Casari C, et al A major role for side-chain polyglutamine hydrogen bonding in irreversible ataxin-3 aggregation. Buckle AM, editor. PLoS One. Public Library of Science; 2011;6: 10 Available: http://dx.plos.org/10.1371/journal.pone.0018789 10.1371/journal.pone.0018789 PMC3076451 21533208

[22] Ellisdon AM, Pearce MC, Bottomley SP. Mechanisms of ataxin-3 misfolding and fibril formation: kinetic analysis of a disease-associated polyglutamine protein. J Mol Biol. 2007;368: 595–605. 10.1016/j.jmb.2007.02.058 17362987

[23] Saunders HM, Gilis D, Rooman M, Dehouck Y, Robertson AL, Bottomley SP. Flanking domain stability modulates the aggregation kinetics of a polyglutamine disease protein. Protein Sci. 2011;20: 1675–81. 10.1002/pro.698 21780213 PMC3218360

[24] Robertson AL, Headey SJ, Saunders HM, Ecroyd H, Scanlon MJ, Carver J a, et al Small heat-shock proteins interact with a flanking domain to suppress polyglutamine aggregation. Proc Natl Acad Sci U S A. 2010;107: 10424–9. 10.1073/pnas.0914773107 20484674 PMC2890844

[25] Marchal S, Shehi E, Harricane M-C, Fusi P, Heitz F, Tortora P, et al Structural instability and fibrillar aggregation of non-expanded human ataxin-3 revealed under high pressure and temperature. J Biol Chem. 2003;278: 31554–63. 10.1074/jbc.M304205200 12766160

[26] Blount JR, Tsou W-L, Ristic G, Burr AA, Ouyang M, Galante H, et al Ubiquitin-binding site 2 of ataxin-3 prevents its proteasomal degradation by interacting with Rad23. Nat Commun. 2014;5: 4638 10.1038/ncomms5638 25144244 PMC4237202

[27] Nicastro G, Menon RP, Masino L, Pastore A. Understanding the plasticity of the ubiquitin-protein recognition code: the josephin domain of ataxin-3 is a diubiquitin binding motif. To be Pubblished.: 10.2210/pdb2jri/pdb. 10.2210/pdb2jri/pdb

[28] Nicastro G, Menon RP, Masino L, Knowles PP, McDonald NQ, Pastore A. The solution structure of the Josephin domain of ataxin-3: structural determinants for molecular recognition. Proc Natl Acad Sci U S A. 2005;102: 10493–8. 10.1073/pnas.0501732102 16020535 PMC1180765

[29] Mao Y, Senic-Matuglia F, Di Fiore PP, Polo S, Hodsdon ME, De Camilli P. Deubiquitinating function of ataxin-3: insights from the solution structure of the Josephin domain. Proc Natl Acad Sci U S A. 2005;102: 12700–5. 10.1073/pnas.0506344102 16118278 PMC1188261

[30] Satoh T, Sumiyoshi A, Yagi-Utsumi M, Sakata E, Sasakawa H, Kurimoto E, et al Mode of substrate recognition by the Josephin domain of ataxin-3, which has an endo-type deubiquitinase activity. FEBS Lett. 2014;588: 4422–30. 10.1016/j.febslet.2014.10.013 25448680

[31] Sanfelice D, De Simone A, Cavalli A, Faggiano S, Vendruscolo M, Pastore A. Characterization of the conformational fluctuations in the Josephin domain of ataxin-3. Biophys J. 2014;107: 2932–40. 10.1016/j.bpj.2014.10.008 25517158 PMC4269769

[32] Narayanan C, Weinstock DS, Wu K-P, Baum J, Levy RM. Investigation of the Polymeric Properties of α-Synuclein and Comparison with NMR Experiments: A Replica Exchange Molecular Dynamics Study. J Chem Theory Comput. 2012;8: 3929–3942. 10.1021/ct300241t 23162382 PMC3496295

[33] Wu K-P, Weinstock DS, Narayanan C, Levy RM, Baum J. Structural reorganization of alpha-synuclein at low pH observed by NMR and REMD simulations. J Mol Biol. 2009;391: 784–96. 10.1016/j.jmb.2009.06.063 19576220 PMC2766395

[34] Wei G, Shea J-E. Effects of solvent on the structure of the Alzheimer amyloid-beta(25–35) peptide. Biophys J. 2006;91: 1638–47. 10.1529/biophysj.105.079186 16766615 PMC1544315

[35] De Simone A, Kitchen C, Kwan AH, Sunde M, Dobson CM, Frenkel D. Intrinsic disorder modulates protein self-assembly and aggregation. Proc Natl Acad Sci U S A. 2012;109: 6951–6. 10.1073/pnas.1118048109 22509003 PMC3344965

[36] Baumketner A, Shea J-E. Folding landscapes of the Alzheimer amyloid-beta(12–28) peptide. J Mol Biol. 2006;362: 567–79. 10.1016/j.jmb.2006.07.032 16930617

[37] Laio A, Gervasio FL. Metadynamics: a method to simulate rare events and reconstruct the free energy in biophysics, chemistry and material science. Reports on Progress in Physics. 2008 p. 126601 10.1088/0034-4885/71/12/126601

[38] Spiwok V, Lipovová P, Králová B. Metadynamics in essential coordinates: free energy simulation of conformational changes. J Phys Chem B. 2007;111: 3073–6. 10.1021/jp068587c 17388445

[39] Limongelli V, Bonomi M, Parrinello M. Funnel metadynamics as accurate binding free-energy method. Proc Natl Acad Sci U S A. 2013;110: 6358–6363. 10.1073/pnas.1303186110 23553839 PMC3631651

[40] Granata D, Camilloni C, Vendruscolo M, Laio A. Characterization of the free-energy landscapes of proteins by NMR-guided metadynamics. Proc Natl Acad Sci U S A. 2013;110: 6817–22. 10.1073/pnas.1218350110 23572592 PMC3637744

[41] Sutto L, Marsili S, Gervasio FL. New advances in metadynamics. Wiley Interdiscip Rev Comput Mol Sci. 2012;2: 771–779. 10.1002/wcms.1103

[42] Nicastro G, Habeck M, Masino L, Svergun DI, Pastore A. Structure validation of the Josephin domain of ataxin-3: conclusive evidence for an open conformation. J Biomol NMR. 2006;36: 267–77. 10.1007/s10858-006-9092-z 17096206

[43] Apicella A, Soncini M, Deriu MA, Natalello A, Bonanomi M, Dellasega D, et al A hydrophobic gold surface triggers misfolding and aggregation of the amyloidogenic Josephin domain in monomeric form, while leaving the oligomers unaffected. PLoS One. Public Library of Science; 2013;8: e58794 10.1371/journal.pone.0058794 23527026 PMC3602447

[44] Hornak V, Abel R, Okur A, Strockbine B, Roitberg A, Simmerling C. Comparison of multiple Amber force fields and development of improved protein backbone parameters. Proteins. 2006;65: 712–25. 10.1002/prot.21123 16981200 PMC4805110

[45] Lindorff-Larsen K, Piana S, Palmo K, Maragakis P, Klepeis JL, Dror RO, et al Improved side-chain torsion potentials for the Amber ff99SB protein force field. Proteins. 2010;78: 1950–8. 10.1002/prot.22711 20408171 PMC2970904

[46] Lindorff-Larsen K, Maragakis P, Piana S, Eastwood MP, Dror RO, Shaw DE. Systematic validation of protein force fields against experimental data. PLoS One. 2012;7: e32131 10.1371/journal.pone.0032131 22384157 PMC3285199

[47] Jorgensen WL, Chandrasekhar J, Madura JD, Impey RW, Klein ML. Comparison of simple potential functions for simulating liquid water. J Chem Phys. 1983;79: 926 10.1063/1.445869

[48] Bussi G, Donadio D, Parrinello M. Canonical sampling through velocity rescaling. J Chem Phys. AIP; 2007;126: 014101 10.1063/1.2408420 17212484

[49] Berendsen HJC, Postma JPM, Van Gunsteren WF, DiNola A, Haak JR. Molecular dynamics with coupling to an external bath. J Chem Phys. AIP; 1984;81: 3684–3690. 10.1063/1.448118

[50] Sun X, Cheng J, Wang X, Tang Y, Ågren H, Tu Y. Residues remote from the binding pocket control the antagonist selectivity towards the corticotropin-releasing factor receptor-1. Sci Rep. 2015;5: 8066 10.1038/srep08066 25628267 PMC4308710

[51] Sun H, Li Y, Tian S, Wang J, Hou T. P-loop Conformation Governed Crizotinib Resistance in G2032R-Mutated ROS1 Tyrosine Kinase: Clues from Free Energy Landscape. Briggs JM, editor. PLoS Comput Biol. 2014;10: e1003729 10.1371/journal.pcbi.1003729 25033171 PMC4102447

[52] Manara RMA, Jayne Wallace E, Khalid S. DNA sequencing with MspA: Molecular Dynamics simulations reveal free-energy differences between sequencing and non-sequencing mutants. Sci Rep. 2015;5: 12783 10.1038/srep12783 26255609 PMC4530457

[53] Hess B, Kutzner C, van der Spoel D, Lindahl E. GROMACS 4: Algorithms for Highly Efficient, Load-Balanced, and Scalable Molecular Simulation. J Chem Theory Comput. 2008;4: 435–447. 10.1021/ct700301q 26620784

[54] Humphrey W, Dalke A, Schulten K. VMD: visual molecular dynamics. J Mol Graph. 1996;14: 33–8, 27–8. Available: http://www.ncbi.nlm.nih.gov/pubmed/8744570 8744570 10.1016/0263-7855(96)00018-5

[55] Heinig M, Frishman D. STRIDE: a web server for secondary structure assignment from known atomic coordinates of proteins. Nucleic Acids Res. 2004;32: W500–W502. 10.1093/nar/gkh429 15215436 PMC441567

[56] Maisuradze GG, Liwo A, Scheraga H a. Principal component analysis for protein folding dynamics. J Mol Biol. Elsevier Ltd; 2009;385: 312–29. 10.1016/j.jmb.2008.10.018 18952103 PMC2652707

[57] Bonomi M, Branduardi D, Bussi G, Camilloni C, Provasi D, Raiteri P, et al PLUMED: A portable plugin for free-energy calculations with molecular dynamics. Comput Phys Commun. 2009;180: 1961–1972. 10.1016/j.cpc.2009.05.011

[58] Barducci A, Bonomi M, Parrinello M. Metadynamics. Wiley Interdisciplinary Reviews: Computational Molecular Science. 2011 pp. 826–843. 10.1002/wcms.31

[59] Laio A, Parrinello M. Escaping free-energy minima. Proc Natl Acad Sci U S A. 2002;99: 12562–12566. 10.1073/pnas.202427399 12271136 PMC130499

[60] Kumar S, Rosenberg JM, Bouzida D, Swendsen RH, Kollman PA. THE weighted histogram analysis method for free-energy calculations on biomolecules. I. The method. J Comput Chem. 1992;13: 1011–1021. 10.1002/jcc.540130812

[61] Bonomi M, Barducci A, Parrinello M. Reconstructing the equilibrium boltzmann distribution from well-tempered metadynamics. J Comput Chem. 2009;30: 1615–1621. 10.1002/jcc.21305 19421997

[62] Formoso E, Limongelli V, Parrinello M. Energetics and Structural Characterization of the large-scale Functional Motion of Adenylate Kinase. Sci Rep. 2015;5: 8425 10.1038/srep08425 25672826 PMC4325324

[63] Berteotti A, Cavalli A, Branduardi D, Gervasio FL, Recanatini M, Parrinello M. Protein Conformational Transitions: The Closure Mechanism of a Kinase Explored by Atomistic Simulations. J Am Chem Soc. 2009;131: 244–250. 10.1021/ja806846q 19067513

[64] Barducci A, Bonomi M, Prakash MK, Parrinello M. Free-energy landscape of protein oligomerization from atomistic simulations. Proc Natl Acad Sci. 2013;110: E4708–E4713. 10.1073/pnas.1320077110 24248370 PMC3856838

[65] Drinkwater N, Cossins BP, Keeble AH, Wright M, Cain K, Hailu H, et al Human immunoglobulin E flexes between acutely bent and extended conformations. Nat Struct Mol Biol. 2014;21: 397–404. 10.1038/nsmb.2795 24632569 PMC3977038

[66] Sicard F, Senet P. Reconstructing the free-energy landscape of Met-enkephalin using dihedral principal component analysis and well-tempered metadynamics. J Chem Phys. 2013;138: 235101 10.1063/1.4810884 23802984

[67] Leone V, Lattanzi G, Molteni C, Carloni P. Mechanism of Action of Cyclophilin A Explored by Metadynamics Simulations. Pettitt BM, editor. PLoS Comput Biol. 2009;5: e1000309 10.1371/journal.pcbi.1000309 19282959 PMC2643488

[68] Zhang Y, Niu H, Li Y, Chu H, Shen H, Zhang D, et al Mechanistic insight into the functional transition of the enzyme guanylate kinase induced by a single mutation. Sci Rep. 2015;5: 8405 10.1038/srep08405 25672880 PMC4325336

[69] Spiwok V, Oborský P, Pazúriková J, Křenek A, Králová B. Nonlinear vs. linear biasing in Trp-cage folding simulations. J Chem Phys. 2015;142: 115101 10.1063/1.4914828 25796266

