# Supplementary material for: Josephin Domain Structural Conformations Explored by Metadynamics in Essential Coordinates
Source: PLoS Comput Biol. 2016 Jan 8;12(1):e1004699. doi: 10.1371/journal.pcbi.1004699 (PMC4706304; doi:10.1371/journal.pcbi.1004699)
Supplement: S1 Text — (PDF) [file pcbi.1004699.s001.pdf]

S1 Text

Section 1

Classical Molecular Dynamics and Metadynamics Lowest Energy States

Five different replicas of the Josephin Domain (JD) in explicitly modeled water were simulated by classical Molecular Dynamics (MD) for 500 ns. In Figure S1 the evolution of the C_α-C_α RMSD relative to the average JD structure (calculated by averaging the last 100 ns of each MD simulation) is reported for each MD replica, in order to check if the system conformation is stable under simulation. The RMSD value results reasonably stable in the last 100 ns of each simulation (Figure S1).

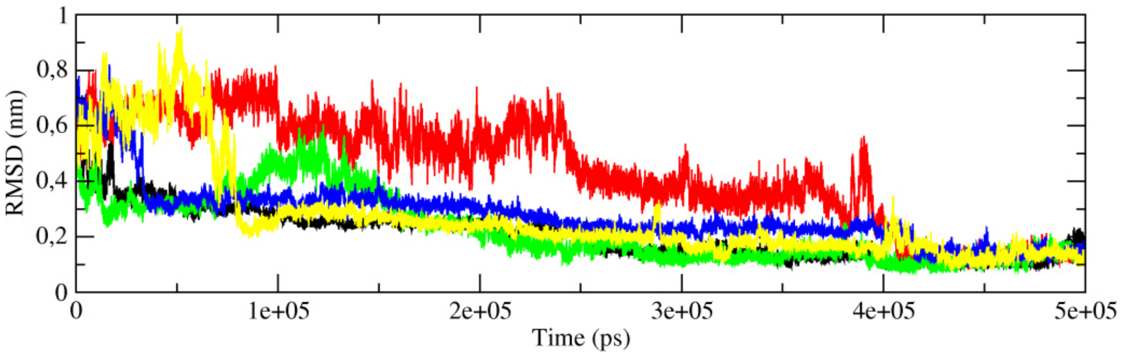

Figure S1. Time evolution of theC_α-C_αRoot Mean Square Deviation (RMSD) relative to the average structure (calculated by averaging the last 100 ns of each MD simulation). The RMSD plot is represented for each MD replica.

Figure S2 shows the JD's secondary structure probability, calculated over the available NMR configurations contained in 1YZB, 2AGA, 2DOS, 2JRI, several configurations at the lowest energy state coming from metadynamics and the overall MD trajectory in the last 100 ns. MD data coming from the five replicas are used as an ensemble. The JD secondary structure showed to be reasonably conserved among all NMR structures, metadynamics and classical MD simulations.

Protein-protein contacts (Figure S3) have been also investigated with particular attention to hydrophobic contacts for available NMR data and metadynamics lowest energy states. Contact matrixes

appear to be similar among all considered cases. Nevertheless, it is worth noticing how half-closed and closed structures (2DOS, 2AGA and metadynamics lowest energy state) share similar contact areas which are not present in 1YZB and 2JRI. Those areas are: α3, a region between β1 and α5 and β6 (Figure S2).

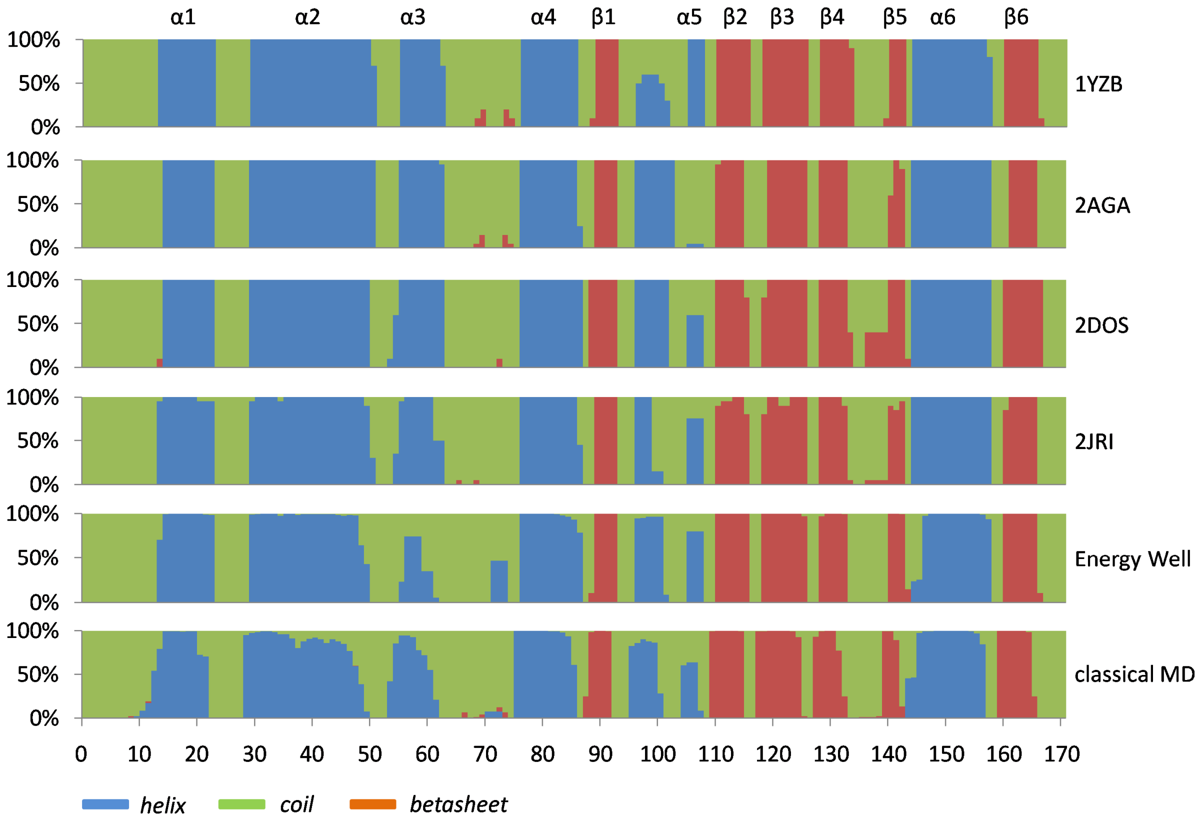

Figure S2. Secondary structure percentage, calculated over the available NMR configurations contained in 1YZB, 2AGA, 2DOS, 2JRI, several configurations at the lowest energy state coming from metadynamics and the overall MD trajectory in the last 100 ns for the simulated five replicas. The alpha-helix (blue), beta-sheet (orange) and unstructured coil (green) are represented.

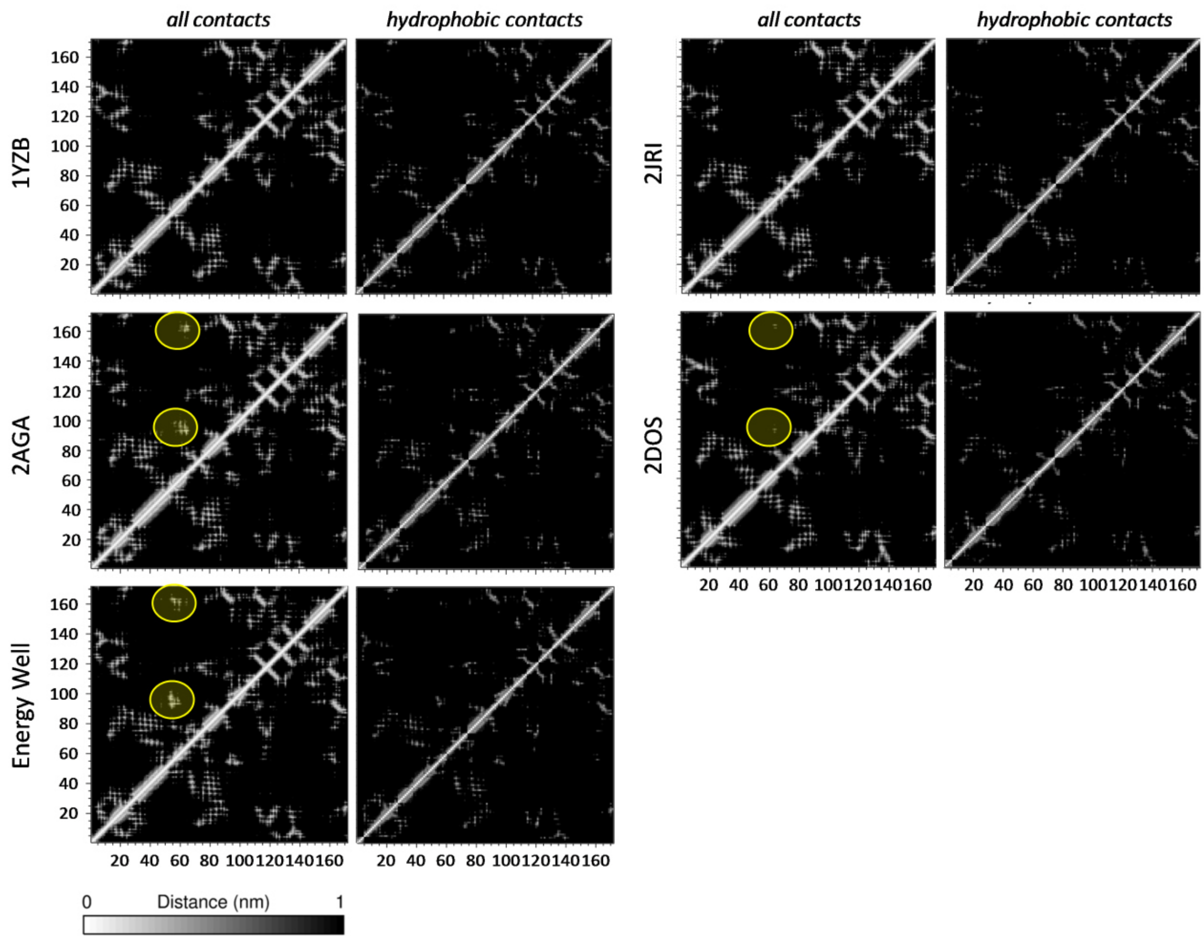

Figure S3. Protein-Protein contacts matrix and Protein-Protein hydrophobic contacts matrix (all heavy atoms have been considered), calculated as average over the available NMR configurations contained in 1YZB, 2AGA, 2DOS, 2JRI, and several configurations at the lowest energy state coming from the metadynamics simulation. In yellow are highlighted similar contacts in lowest energy states, half-closed and closed NMR structures.

Section 2

Principal Component Analysis

Principal Component Analysis (PCA) was applied to the whole data coming from the MD trajectory pertaining to the five replicas used as ensemble in order to provide collective motion directly related to the hairpin closure. In detail, after the alignment of the protein *Cα* positions, the covariance matrix was calculated and diagonalized.

The visualization of the first 10 eigenvalues (Figure S4), representative of the variance of the principal components, clearly highlights that the first eigenvalue accounts for the 50 % of the total variance in the data set.

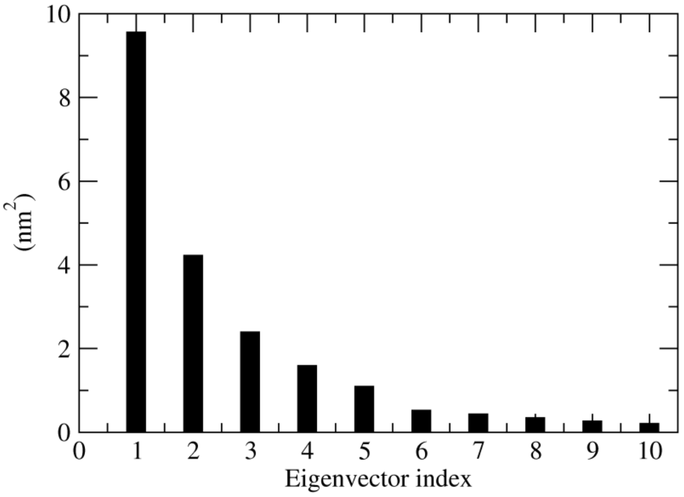

Figure S4. Eigenvalues obtained applying the PCA to the classical MD trajectory.

Section 3

Metadynamics Collective Variables

The estimation of the free energy profile was performed by employing the reweighted-histogram procedure [1,2], taking into account four collective variables: the projection along the first PCA eigenvector, the JD Radius of Gyration (RG), the hairpin angle [3] and the alphaRMSD variable (parameters r_0=0.08 nm, n=6, and m=12).

PCA eigenvector

The first eigenvector derived from the PCA was used as CV for a well-tempered Metadynamics simulation of 500 ns starting from the open-like 1YZB model [4]. To perform Metadynamics, a Gaussian width of 0.1 was used. Along the simulation, the initially Gaussian deposition rate value of 0.2 kJ/mol·ps was used and it gradually decreased on the basis of an adaptive scheme, with a bias factor of 20. The Gaussian width value was the same order of magnitude as the standard deviation of the CVs (Table 1), calculated during unbiased simulations (production MD).

Table 1. Standard deviation of the projection along the first PCA vector calculated during the unbiased MD simulations.

UNBIASED MD SIMULATION	STANDARD DEVIATION OF THE PROJECTION ALONG THE FIRST PCA VECTOR
**1**	0.13
**2**	0.7
**3**	0.5
**4**	0.11
**5**	0.22

Radius of Gyration

The JD Cα-Radius of Gyration (RG), already used in previous studies [3,5] to analyze the JD conformational transition, was considered as second CV. Given that the NMR models (1YZB, 2JRI, 2DOS, and 2AGA) considered in this work present a different number of residues, the RG has been calculated by considering all the residues in common among the above mentioned PDB models. In detail, the residue range 1Met-171Asp (according to 1YZB numbering) has been chosen.

AlphaRMSD

The alphaRMSD variable represents the distance from the alpha helix configurations to measure the number of segments that have an alpha helical configuration. This is done by calculating the following sum of functions of the RMSD distances:

$$s = \sum_i \frac{1 - \left(\frac{r_i - d_0}{r_0}\right)^n}{1 - \left(\frac{r_i - d_0}{r_0}\right)^m}$$

where the sum runs over all possible segments of alpha helix.

Angle

As third reaction coordinate we selected a large-scale bending angle between the globular and the helical hairpin subdomain. This reaction coordinate has been previously used in a recent literature [3] to estimate the JD free energy profile. The angle is here calculated from the centers of mass of the *Cα* atoms from three distinct JD regions (Figure S5): globular subdomain (residues 111-113, 122-125 and 162-165), hinge (residues 32-35) and loop (residues 45-48 and 58-61) [3].

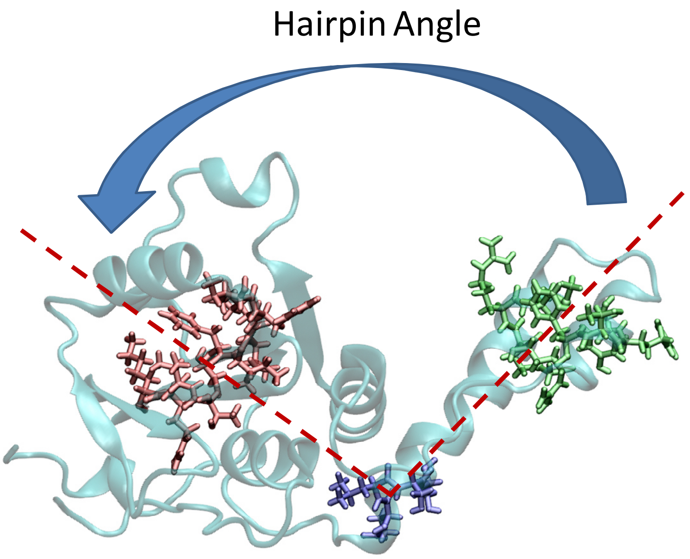

Figure S5. The hairpin angle is defined from the centers of mass of the *Cα* atoms from three distinct JD regions: globular subdomain (highlighted in red), hinge (highlighted in violet) and loop (highlighted in green).

Convergence of the free energy estimation

The convergence of the Metadynamics simulation was demonstrated by following a well established computational procedure performed in a recent work [6], which requires to check *i*) recrossing events between low energy states and *ii*) fluctuations of the free energy difference, around a specific value.

i) Several recrossing events between low energy states can be identified analyzing the time evolution of the hairpin angle and radius of gyration (Figure S6). These events lead to the convergence in the estimation of the protein free energy state, as reported in Figure S7.

ii) The free energy difference between low energy states at different times along the simulation (Figure S7) was calculated to assess the convergence. The Figure S7 shows that the free energy difference is reasonably stable from 100 to 500 ns. The uncertainty, calculated as the SD from the asymptotic value of the free energy obtained from the last part of the simulation is 0.5 kJ/mol.

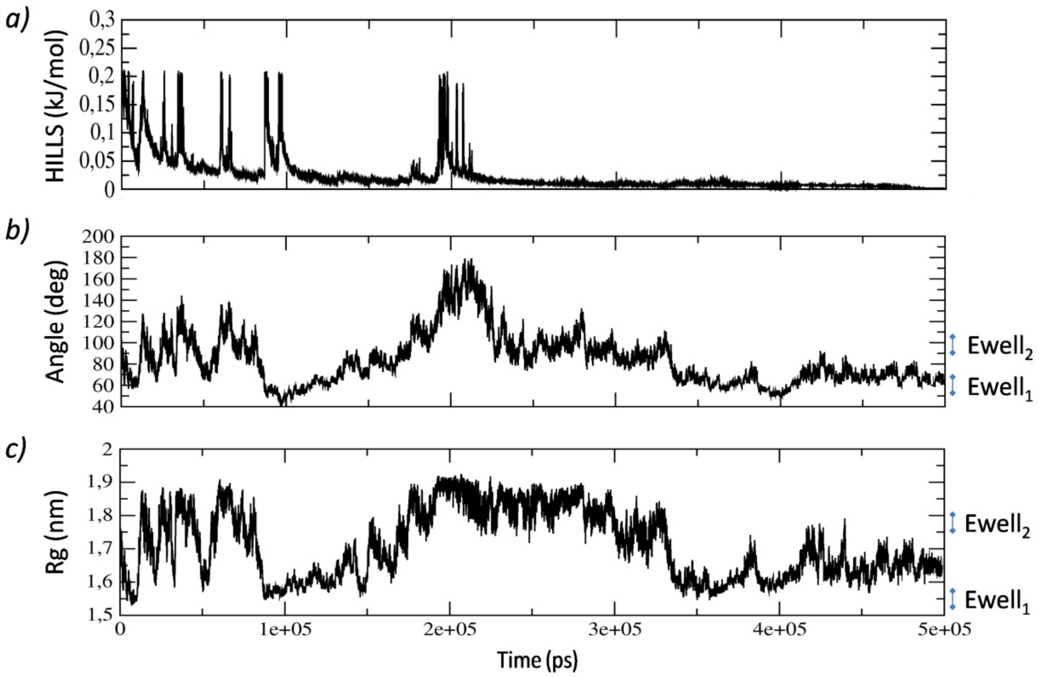

Figure S6. a) Plot of the Gaussian Height added to the system along the Metadynamics simulation. Several recrossing events between the low energy states can be identified, even when the added Gaussian becomes very small. These events lead to the convergence in the estimation of the JD free energy profile.

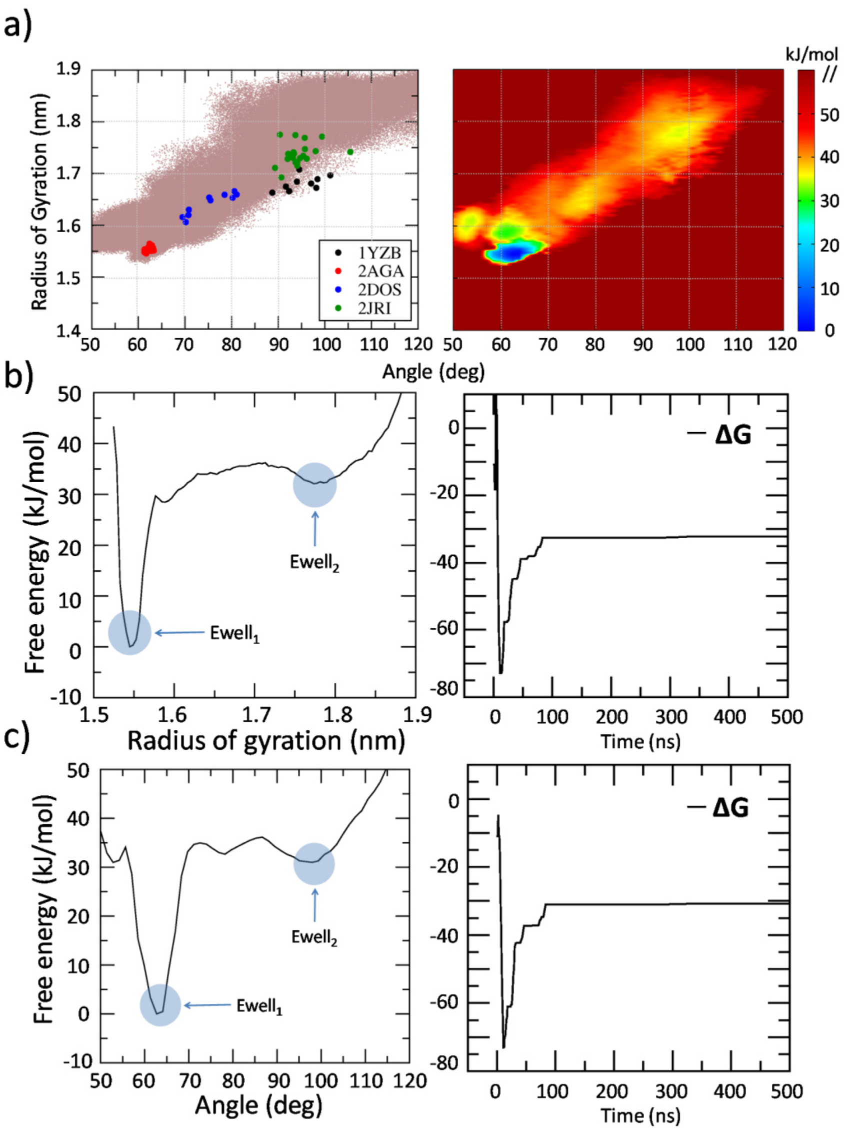

Figure S7. a) (Left) Scatter plot of the Radius of Gyration vs Hairpin Angle, collecting all of the configurations sampled during the Metadynamics simulations. (Right) Representation of the Free Energy Surface computed as function of the JD radius of gyration and the hairpin angle by applying a reweighting algorithm. b) (Left) Free Energy profile as function of the JD Radius of Gyration obtained by reweighting the Metadynamics data. The Energy wells are highlighted in blue. (Right) Convergence of the Free Energy calculation. ΔG is calculated using 1.54 < Ewell1 < 1.56 nm and 1.77 < Ewell2 < 1.79 nm. c) (Left) Free Energy profile as function of the hairpin angle obtained by reweighting the Metadynamics data. The Energy wells are highlighted in blue. (Right) Convergence of the Free Energy calculation. ΔG is calculated using 62 < Ewell1 < 64 ° and 97 < Ewell2 < 99 °. The uncertainty, calculated as the SD from the asymptotic value of the free energy obtained from the last part of the simulation is 0.5 kJ/mol.

References

1. Kumar S, Rosenberg JM, Bouzida D, Swendsen RH, Kollman PA. THE weighted histogram analysis method for free-energy calculations on biomolecules. I. The method. J Comput Chem. 1992;13: 1011–1021. doi:10.1002/jcc.540130812

2. Bonomi M, Barducci A, Parrinello M. Reconstructing the equilibrium boltzmann distribution from well-tempered metadynamics. J Comput Chem. 2009;30: 1615–1621. doi:10.1002/jcc.21305

3. Sanfelice D, De Simone A, Cavalli A, Faggiano S, Vendruscolo M, Pastore A. Characterization of the conformational fluctuations in the Josephin domain of ataxin-3. Biophys J. 2014;107: 2932–40. doi:10.1016/j.bpj.2014.10.008

4. Masino L, Nicastro G, Menon RP, Dal Piaz F, Calder L, Pastore A. Characterization of the structure and the amyloidogenic properties of the Josephin domain of the polyglutamine-containing protein ataxin-3. J Mol Biol. 2004;344: 1021–35. doi:10.1016/j.jmb.2004.09.065

5. Deriu MA, Grasso G, Licandro G, Danani A, Gallo D, Tuszynski JA, et al. Investigation of the josephin domain protein-protein interaction by molecular dynamics. Salahub D, editor. PLoS One. Public Library of Science; 2014;9: e108677. doi:10.1371/journal.pone.0108677

6. Limongelli V, Bonomi M, Parrinello M. Funnel metadynamics as accurate binding free-energy method. Proc Natl Acad Sci U S A. 2013;110: 6358–6363. doi:10.1073/pnas.1303186110
